# Retraction: Nonalcoholic fatty liver disease, diet and gut microbiota

**Published:** 2017-10-18

**Authors:** Carmine Finelli, Giovanni Tarantino

**Affiliations:** 1 Center of Obesity and Eating Disorders, Stella Maris Mediterraneum Foundation, C/da S. Lucia, Chiaromonte, 80035 Potenza, Italy; 2 Department of Clinical Medicine and Surgery, Federico II University Medical School of Naples, Via Sergio Pansini, 5, 80131Naples, Italy; 3 National Cancer Institute "Foundation G. Pascale" -IRCS- 83013 Mercogliano (Av), Italy

Dear editor,

As corresponding author I ask for retraction of our article Finelli and Tarantino, 2014, *EXCLI J* 2014;13: 461 because parts of it have been published previously. Both authors agree to this retraction. The authors apologize for any inconvenience to the scientific community.

Giovanni Tarantino

